# Lens opacity as a predictor of retinal vasculature change following cataract surgery

**DOI:** 10.1038/s41598-025-19037-z

**Published:** 2025-09-25

**Authors:** Lars H. B. Mackenbrock, An Ting L. Xu, Grzegorz Łabuz, Victor A. Augustin, Timur M. Yildirim, Gerd U. Auffarth, Ramin Khoramnia

**Affiliations:** 1 Department of Ophthalmology, Faculty of Medicine and University Hospital Carl Gustav Carus, TU Dresden, Fetscherstraße 74, 01307 Dresden, Germany; 2 Department of Ophthalmology, University Hospital Heidelberg, Heidelberg, Germany; 3 Department of Ophthalmology, University Hospital Mannheim, Mannheim, Germany

**Keywords:** Medical research, Lens diseases, Retinal diseases

## Abstract

Cataract surgery, one of the most common surgical procedures worldwide, significantly improves visual acuity and quality of life for patients. However, recent studies suggest that it may have broader implications for ocular health, including changes in retinal perfusion. This prospective clinical study investigates the relationship between preoperative lens opacity and postoperative changes in macular perfusion using optical coherence tomography in 46 patients. Objective metrics were assessed automatically using a custom computer script. The analysis revealed significant increases in vessel density, diameter, and complexity across the superficial, intermediate, and deep retinal vascular plexuses, with the most pronounced changes occurring within the first postoperative week. A strong correlation was observed between preoperative nuclear lens opacity and the increase in macular perfusion, suggesting that reduced light transmission through dense cataracts may drive postoperative functional hyperemia. In contrast, surgical parameters such as phacoemulsification energy showed no significant association, and intraocular pressure reduction correlated only with subtle vascular perimeter changes. These findings indicate that enhanced light exposure following cataract removal—rather than just inflammation or mechanical factors—likely stimulates adaptive retinal metabolic responses. Clinically, this highlights the importance of preoperative lens opacity assessment as a predictor of vascular remodeling, potentially informing strategies to mitigate complications in the short and long term.

## Introduction

Cataract surgery is one of the most commonly performed surgical procedures worldwide, offering significant improvements in visual acuity and quality of life for patients^1^. However, recent studies have shown that the procedure may have broader implications for ocular health. Changes in retinal perfusion can potentially lead to both short-term complications such as the pseudophakic cystoid macular edema (PCME), as well as long-term complications, such as the progression of age-related macular degeneration (AMD), or increase the risk of retinal vein occlusion^2^. While it is known that cataract surgery induces an increase in macular perfusion, the etiology behind this change is unknown^3–5^. The present study aims to investigate the relationship between the extent of lens opacity and the postoperative changes in retinal vasculature following cataract surgery. Optical coherence tomography angiography (OCTA) has emerged as a non-invasive imaging technique that allows for detailed visualization and quantification of retinal vasculature, while anterior segment OCT (AS-OCT) enables high resolution imaging and quantification of the crystalline lens. This prospective study quantifies changes in the superficial vascular plexus (SVP), intermediate capillary plexus (ICP), and deep capillary plexus (DCP) using objective indices. By correlating these changes with preoperative lens opacity measurements and surgical parameters, we aim to explore the potential predictive value of lens opacity for postoperative retinal vascular changes.

## Methods

### Setting and patients

In this prospective study we enrolled forty-six eyes from forty-six patients scheduled for routine cataract surgery at our centre. The study included adult patients with senile, traumatic, or iatrogenic cataracts. Exclusion criteria were a spherical equivalent refraction greater than 8 diopters, a history of previous ocular surgery, any ocular conditions (such as glaucoma, uveitis, retinal disorders, central corneal opacities, etc.), or systemic disorders (such as diabetes or vascular diseases) that could confound the analysis. Only one eye per patient was included. For patients requiring surgery on both eyes, the eye with the highest preoperative OCTA quality was selected. All patients were examined before surgery (T0), as well as 1 week (T1), 4 weeks (T2) and 12 weeks (T3) post-operatively. Each follow-up visit included Snellen corrected distance visual acuity (CDVA), slit-lamp microscopy, Goldman applanation tonometry and a comprehensive fundus examination. The study received approval from the ethics committee of the medical faculty of the Ruprecht Karls University Heidelberg (S-644/2020) and was conducted in accordance with the Declaration of Helsinki, with written informed consent obtained from all participants.

### Cataract opacity measurement

**Fig. 1:**
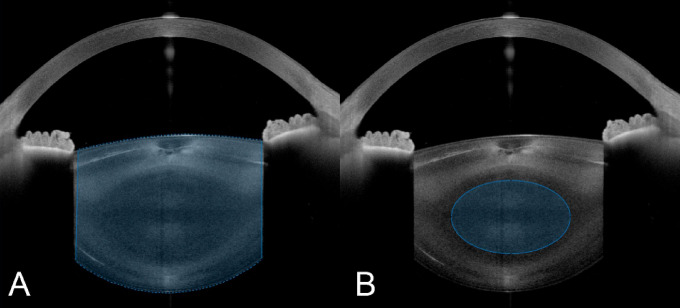
Anterior segment optical coherence tomography scan of the crystalline lens with the segmentation of the entire lens (**A**) and the nucleus (**B**).

Objective cataract opacity measurement was achieved using anterior segment optical coherence tomography (AS-OCT). Each eye was scanned with the ANTERION (Heidelberg Engineering GmbH, Heidelberg, Germany), a swept-source OCT (SS-OCT) device capable of producing high-resolution cross-sectional images of the anterior segment. A radial pattern consisting of 15 B-scans was used to include local anatomical variations in the lens. Beforehand, full mydriasis was achieved in all patients with phenylephrine 5% and tropicamide eye drops in order to visualize as much of the crystalline lens as possible. Images that failed to meet the inbuilt quality standards due to blinking, poor alignment, or poor fixation were excluded, and the examination was repeated. The AS-OCT images were analyzed using a custom MATLAB (Version R2021b, MathWorks, Natick, MA, USA) script using the following method: The images were binarized using a general threshold. The approximate shape of the anterior and posterior lens surfaces resembles a 4th-order polynomial curve. The script calculates the 4th-order polynomial that best fits the detected edge and corrects substantially deviating points, allowing it to justify local segmentation errors caused by artifacts or signal falloff. The mean pixel intensity (0 to 255 scale) of the segmented area represents the lens opacity (encompassing the nucleus and cortex) (Fig. 1A). To account for pixel intensity variations between individual B-scans due to anatomical differences and uneven illumination, the final lens density is derived by averaging all 15 B-Scans. To calculate the opacity of the lens nucleus (nuclear opacity), the script positions an elliptical region of interest (ROI) at the center of the previously segmented lens, and if necessary, its size and position is manually corrected (Fig. 1B). Two independent, experienced observers reviewed all segmentations to detect any errors in the automatic segmentation. This method has been validated in a previous study by our group^6–9^.

### Macular perfusion analysis

Macular perfusion was quantified with optical coherence tomography angiography (OCTA) using the SPECTRALIS (Heidelberg Engineering GmbH, Heidelberg, Germany), a spectral-domain optical coherence tomography (SD-OCT) device. The simultaneous registration of OCT and confocal scanning laser ophthalmoscopy (cSLO) images allows follow-up examinations to be at the exact same position, enabling a precise comparison of retinal vasculature over an extended period of time^10^. A 3 × 3 mm square centered on the fovea was used, with all OCTA scans performed by a single experienced operator. Images with a quality index < 30 were discarded, and the scan was repeated. To increase the image quality, the automatic real time mean (ART) algorithm built into the SPECTRALIS was implemented: by averaging multiple B-Scans, the signal-to-noise ratio of the image is continuously increased by the square root of the number of B-Scans, leading to improved contrast and reduced speckle pattern, enabling a clearer signal, even with dense cataracts^10^.

**Fig. 2:**
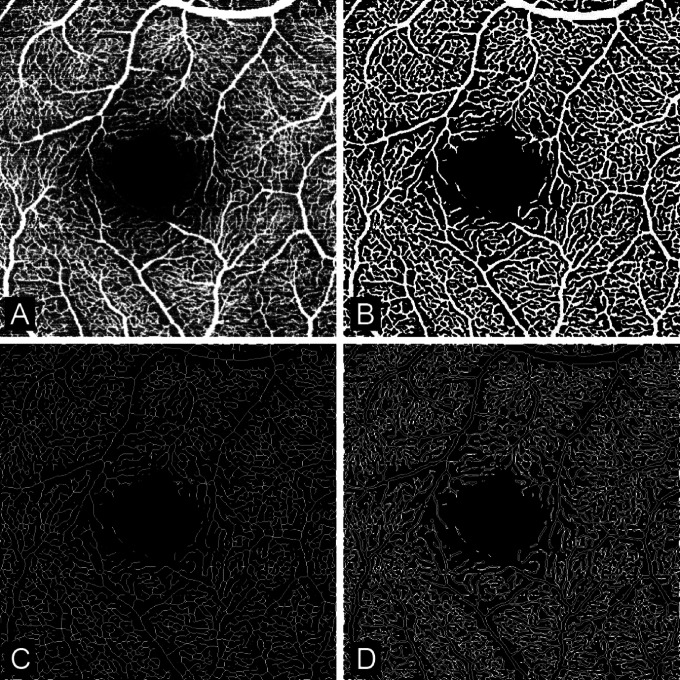
Overview of the different OCTA images used to calculate the perfusion metrics. A is the raw OCTA scan, B the binarized imaged, C the skeletonized image and D the perimeter image.

The different vascular plexus of the retina can be segmented automatically using the in-build software of the SPECTRALIS. In this study, we analyzed the superficial vascular plexus (SVP) located in the ganglion cell layer and in the inner part of the inner plexiform layer, the intermediate capillary plexus (ICP) located in the outer part of the inner plexiform layer and the inner part of the inner nuclear layer, and the deep capillary plexus (DCP) located in the outer part of the inner nuclear layer and in the outer plexiform layer. The automatic segmentations were verified by an expert, corrected if necessary, and the generated en-face images (Fig. 2A) were exported as lossless PNG files. The analysis of the OCTA images was again done using a custom written MATLAB script. First, a Frangi filter was applied to the greyscale image to enhance the vessels. The filter works by analyzing the Hessian matrix of the image at multiple scales, computing eigenvalues to assess local curvature, and calculating a “vesselness” measure for each pixel, effectively highlighting tubular structures while suppressing background noise^11^. Then, the image was binarized (Fig. 2B) using a general threshold calculated using Otsu’s method^12^. This binarization method has been validated in previous studies^13^.

The binary image can then be used to calculate different metrics to quantitatively analyze the OCTA scans. While countless metrics have been introduced in the last decade^14,15^, we opted to implement the most frequent, which are briefly described here. In the following, *x* and *y* are the coordinates of each pixel and *n* the total amount of pixels in the OCTA image. The vessel area density (VAD) is the ratio between the area occupied by blood vessels and the total area of the image and is defined as

1 $$VAD=\frac{{\sum\:}_{x=1,\:\:y=1}^{n}A\left(x,y\right)}{{\sum\:}_{x=1,\:\:y=1}^{n}I\left(x,y\right)}$$

where A represents the pixels occupied by a vessel (white pixels in Fig. 2B) and I is all the pixels in the image. The vessel diameter index (VDI) is the ratio between vessel area and the vessel length and is used to quantify vascular dilation or shrinkage. It is defined as

2 $$\:\begin{array}{c}VDI=\frac{{\sum\:}_{x=1,\:\:y=1}^{n}A\left(x,y\right)}{{\sum\:}_{x=1,\:\:y=1}^{n}S\left(x,y\right)}\end{array}$$

where A represents the pixels occupied by a vessel (white pixels in Fig. 2B) and S the pixels occupied in the skeletonized image (i.e. the overall vessel length), which is obtained from an algorithm that reduces the vessel structures to single-pixel-wide lines while preserving their connectivity (Fig. 2C). The skeletonized image can also be used to calculate the total amount of endpoints (EP), the total amount of branchpoints (BP), the total vessel length (TVL) and the average vessel length (AVL). The vessel perimeter index (VPI) is calculated as the ratio of the vessel perimeter to the total blood vessel area and can be a sign of vessel dropout and early ischemia. It is defined as

3 $$\:\begin{array}{c}VPI=\frac{{\sum\:}_{x=1,\:\:y=1}^{n}P\left(x,y\right)}{{\sum\:}_{x=1,\:\:y=1}^{n}A\left(x,y\right)}\end{array}$$

where P represents the pixels occupied by the perimeter of the blood vessel (i.e. the overall contour length, Fig. 2C) and A the pixels occupied by blood vessels (white pixels in Fig. 2B). The blood vessel tortuosity index (BVT) quantifies the extent of vessel distortion and is defined as

4 $$\:\begin{array}{c}BVT=\frac{1}{n}\sum\:_{i=1}^{n}\left(\frac{\left\{Geodesic\:distance\:between\:two\:endpoints\:of\:a\:veseel\:i\right\}}{\left\{Euclidean\:distance\:between\:two\:endpoints\:of\:a\:veseel\:i\right\}}\right)\end{array}$$

where i represents the i^th^ vessel branch and n the total number of branches. The geodesic distance is the real vessel branch length (calculated using the skeletonized image), while the Euclidean distance is the straight-line distance between the two end points of the vessel branch. The efficiency of blood transportation decreases with the degree of vessel tortuosity and is therefore an indicator of vessel health. The vessel complexity index (VCI) quantifies the characteristics of the retinal vasculature, testing their circularity, and is defined as

5 $$VCI=\frac{{\left({\sum\:}_{x=1,y=1}^{n}P\left(x,y\right)\right)}^{2}}{4\pi\:{\sum\:}_{x=1,y=1}^{n}A\left(x,y\right)}$$

where P represents the pixels occupied by the perimeter of the blood vessel (white pixels in Fig. 2C) and A the pixels occupied by blood vessels (white pixels in Fig. 2B). In most cases, less complex vasculature is a result of decreased perfusion.

### Cataract surgery

All patients were operated on by a single experienced surgeon using the divide and conquer technique. Phacoemulsification was performed with the Centurion Vision System (Alcon, Geneva, Switzerland), which automatically displays the cumulative dissipated energy (CDE), the phacoemulsification time and the amount of fluid used. The CDE is traditionally calculated as CDE (%s) = Average phacoemulsification power (%) x Phacoemulsification time (s)^16^. No intraoperative complication occurred. All patients received dexamethasone and Neomycin eyedrops postoperatively. They were administered four times a day for the first week, with a weekly reduction of one drop.

### Statistical analysis

Data analysis was done using SPSS (Version 29, IBM, Chicago, USA). Values are reported as means ± standard deviation. The repeated measure ANOVA test was used to determine the statistical significance of the change between the different follow ups. Data normality was confirmed using the Shapiro-Wilk test. The Maulchy Test was employed to assess the sphericity of the data. If the data was not spherical, the Greenhouse-Geisser correction was applied. A p-value of 0.05 or less was considered significant. The Pearson correlation coefficient was used to assess the correlation between the vascular perfusion indices and the different functional parameters.

## Results

This study included a total of 46 eyes (26 right and 20 left eyes) of 22 women and 24 men with a mean age of 64.98 ± 10.13 years. The CDE used ranged from 0.00 to 9.81%s with a mean of 2.89 ± 1.80%s. The phacoemulsification time ranged from 0 to 456 s with a mean of 213.35 ± 117.81 s and the amount of fluid used ranged from 24 to 70 ml with a mean of 41.91 ± 12.92 ml. The lens density ranged from 27.59 to 63.66 pixel intensity unit (PIU) with a mean of 43.92 ± 6.27 PIU and the nuclear density ranged from 15.92 to 76.50 PIU with a mean of 36.71 ± 13.67 PIU. The intraocular pressure (IOP) had a mean of 16.08 ± 3.26 mmHg at T0, 15.40 ± 4,52 mmHg at T1, 14.29 ± 2.44 at T2 and 12.96 ± 2.95 mmHg at T3. There was a significant difference between the follow-ups, with F = 6.249 and *p* < 0.001. The results of the macular perfusion analysis can be found in Table 1. The results of the correlation analysis between the change of the vasculature parameters and most relevant functional metrics can be found in Table 2. A significant correlation between the change in IOP and vascular metrics could only be established with the VPI in the ICP (*r* = 0.334 with *p* = 0.038) and in the DCP (*r* = 0.433 with *p* = 0.006). No significant correlation was established with the phacoemulsification time or the amount of fluids used.

**Table 1:** Results of the macular perfusion analysis in the superficial vascular plexus (SVP), intermediate capillary plexus (ICP) and deep capillary plexus (DCP). Vessel area density (VAD), vessel diameter index (VDI), vessel perimeter index (VPI), blood vessel tortuosity (BVT), vessel complexity index (VCI), number of branchpoints (BP), total vessel length (TVL), average vessel length (AVL) and number of endpoints (EP) were measured at preoperatively (T0), 1 week (T1), 4 week (T2) and 12 weeks (T3) postoperatively. The repeated measure ANOVA test was used to assess a significant change between the timepoints, with the level of significance p.

Vascular Metric	Vascular Plexus	T0 (Mean ± SD)	T1 (Mean ± SD)	T2 (Mean ± SD)	T3 (Mean ± SD)	ANOVA	*p*
VAD	SVP	0.32 ± 0.04	0.34 ± 0.05	0.34 ± 0.04	0.35 ± 0.03	11.20*	< 0.001
	ICP	0.22 ± 0.04	0.24 ± 0.04	0.23 ± 0.03	0.24 ± 0.02	10.47*	< 0.001
	DCP	0.20 ± 0.04	0.22 ± 0.04	0.22 ± 0.04	0.22 ± 0.03	11.60*	< 0.001
VDI	SVP	3.83 ± 0.18	3.78 ± 0.19	3.77 ± 0.20	3.72 ± 0.16	4.05*	0.009
	ICP	3.06 ± 0.09	3.07 ± 0.09	3.07 ± 0.09	3.06 ± 0.10	0.63	0.594
	DCP	2.64 ± 0.05	2.63 ± 0.06	2.64 ± 0.08	2.62 ± 0.07	1.28	0.283
VPI	SVP	0.70 ± 0.03	0.70 ± 0.02	0.70 ± 0.03	0.71 ± 0.02	1.54	0.207
	ICP	0.91 ± 0.01	0.90 ± 0.01	0.90 ± 0.01	0.90 ± 0.01	3.59*	0.016
	DCP	0.95 ± 0.01	0.95 ± 0.01	0.95 ± 0.01	0.95 ± 0.01	1.03	0.381
BVT	SVP	1.25 ± 0.03	1.26 ± 0.03	1.26 ± 0.03	1.27 ± 0.02	4.19*	0.007
	ICP	1.24 ± 0.02	1.25 ± 0.02	1.25 ± 0.02	1.25 ± 0.02	2.43	0.068
	DCP	1.25 ± 0.02	1.25 ± 0.02	1.25 ± 0.02	1.26 ± 0.02	3.91*	0.016
VCI	SVP	3151 ± 476	3305 ± 504	3369 ± 457	3474 ± 318	8.48*	< 0.001
	ICP	3528 ± 589	3807 ± 575	3797 ± 511	3918 ± 345	9.77*	< 0.001
	DCP	3584 ± 640	3968 ± 685	3930 ± 631	4047 ± 484	12.12*	< 0.001
BP	SVP	1142 ± 345	1329 ± 397	1355 ± 355	1408 ± 289	13.76*	< 0.001
	ICP	628 ± 173	734 ± 170	714 ± 156	752 ± 131	14.12*	< 0.001
	DCP	728 ± 220	900 ± 231	861 ± 218	913 ± 203	16.68*	< 0.001
TVL	SVP	19,465 ± 3127	20,690 ± 3215	21,010 ± 2769	21,592 ± 1884	10.42*	< 0.001
	ICP	15,189 ± 3147	16,784 ± 2981	16,789 ± 2725	17,447 ± 2072	11.36*	< 0.001
	DCP	15,278 ± 3305	17,505 ± 3481	17,367 ± 3282	17,989 ± 2669	15.03*	< 0.001
AVL	SVP	14.70 ± 1.06	14.77 ± 0.97	14.79 ± 0.97	14.84 ± 0.96	0.853	0.439
	ICP	11.97 ± 0.97	12.51 ± 0.90	12.60 ± 0.92	12.76 ± 0.92	12.09*	< 0.001
	DCP	11.23 ± 0.89	11.79 ± 0.91	11.95 ± 0.88	12.03 ± 0.81	15.15*	< 0.001
EP	SVP	1870 ± 306	1766 ± 317	1755 ± 255	1714 ± 329	4.55*	0.007
	ICP	3453 ± 292	3422 ± 402	3389 ± 283	3430 ± 312	0.683	0.526
	DCP	4034 ± 382	4003 ± 518	3931 ± 369	3976 ± 378	0.94	0.422

**Table 2:** Correlation between the different macular perfusion indices and functional metrics. Significant results are marked with *.

Vascular metric	Vascular plexus	Lens opacity	Nuclear opacity	CDE
VAD	SVP	*r* = 0.197 *p* = 0.205	*r* = 0.345* *p* = 0.024	*r* = 0.244 *p* = 0.159
	ICP	*r* = 0.281 *p* = 0.068	*r* = 0.350* *p* = 0.021	*r* = 0.210 *p* = 0.187
	DCP	*r* = 0.299* *p* = 0.050	*r* = 0.303* *p* = 0.010	*r* = 0.157 *p* = 0.326
VDI	SVP	*r* = 0.233 *p* = 0.714	*r* = 0.100 *p* = 0.528	*r* = 0.031 *p* = 0.851
	ICP	*r* = 0.202 *p* = 0.199	*r* = 0.299* *p* = 0.050	*r* = 0.205 *p* = 0.204
	DCP	*r* = 0.233 *p* = 0.137	*r* = 0.339* *p* = 0.028	*r*=-0.023 *p* = 0.886
VPI	SVP	*r*=-0.069 *p* = 0.650	*r*=-0.036 *p* = 0.813	*r*=-0.130 *p* = 0.401
	ICP	*r*=-0.312* *p* = 0.039	*r*=-0.288* *p* = 0.049	*r*=-0.033 *p* = 0.833
	DCP	*r*=-0.199 *p* = 0.196	*r*=-0242. *p* = 0.114	*r*=-0.305* *p* = 0.050
BVT	SVP	*r* = 0.246 *p* = 0.112	*r* = 0.367* *p* = 0.015	*r* = 0.340* *p* = 0.030
	ICP	*r* = 0.080 *p* = 0.606	*r* = 0.098 *p* = 0.528	*r* = 0.106 *p* = 0.504
	DCP	*r* = 0.113 *p* = 0.456	*r* = 0.074 *p* = 0.626	*r*=-0.221 *p* = 0.149
VCI	SVP	*r* = 0.156 *p* = 0.329	*r* = 0.336* *p* = 0.032	*r* = 0.103 *p* = 0.531
	ICP	*r* = 0.257 *p* = 0.092	*r* = 0.370* *p* = 0.014	*r* = 0.224 *p* = 0.154
	DCP	*r* = 0.272 *p* = 0.074	*r* = 0.343* *p* = 0.023	*r* = 0.189 *p* = 0.231
BP	SVP	*r* = 0.132 *p* = 0.400	*r* = 0.282 *p* = 0.067	*r* = 0.268 *p* = 0.090
	ICP	*r* = 0.180 *p* = 0.242	*r* = 0.355* *p* = 0.018	*r* = 0.187 *p* = 0.235
	DCP	*r* = 0.234 *p* = 0.135	*r* = 0.321* *p* = 0.038	*r* = 0.118 *p* = 0.467
TVL	SVP	*r* = 0.265 *p* = 0.085	*r* = 0.332* *p* = 0.030	*r* = 0.265 *p* = 0.094
	ICP	*r* = 0.228 *p* = 0.137	*r* = 0.326* *p* = 0.031	*r* = 0.173 *p* = 0.273
	DCP	*r* = 0.276 *p* = 0.074	*r* = 0.369* *p* = 0.015	*r* = 0.187 *p* = 0.241
AVL	SVP	*r* = 0.376* *p* = 0.011	*r* = 0.326* *p* = 0.029	*r* = 0.300* *p* = 0.050
	ICP	*r* = 0.319* *p* = 0.037	*r* = 0.344* *p* = 0.024	*r* = 0.093 *p* = 0.565
	DCP	*r* = 0.232 *p* = 0.145	*r* = 0.349* *p* = 0.025	*r* = 0.097 *p* = 0.558
EP	SVP	*r*=-0.229 *p* = 0.125	*r*=-0.161 *p* = 0.285	*r*=-0.212 *p* = 0.168
	ICP	*r* = 0.156 *p* = 0.311	*r* = 0.310* *p* = 0.041	*r* = 0.130 *p* = 0.413
	DCP	*r* = 0.228 *p* = 0.137	*r* = 0.242* *p* = 0.023	*r* = 0.330* *p* = 0.033

## Discussion

The current study aims to establish a link between the crystalline lens opacity and the changes in foveal vasculature following cataract surgery. We established that most vascular metrics increase significantly during the first three months after lens exchange. This increase is most pronounced in the first week, and then either reaching a plateau or increasing at a reduced rate in the following months. These results are in line with the work in the literature^3,5,17^. Pilloto et al. showed that the parameters returned to their baseline values^4^. However, this might be due to their small sample size or image analysis method. In a study by Ćurić et al.^17^, it was shown that the parameters remain elevated up to 6 months, demonstrating that cataract surgery has a long-lasting effect on macular perfusion.

The reason behind this increase in perfusion is not fully understood. Inflammation comes to mind as an obvious reason, as cataract surgery leads to a secretion of inflammatory mediators, which diffuse to the posterior segment and impact the blood-retina barrier. This contributes to an increase in foveal capillary permeability and induces further inflammatory cascades. In a rat study by Xu et al., it was shown that extracapsular lens extraction results in an upregulation in IL-1β, CCL2, complement C3, and CFB gene expression in the retina and choroid during a 2-week-long observation period^18^. Comorbidities like diabetes can further intensify the inflammatory response, as illustrated by Ikegami et al.^19^. They found that the aqueous flare value was significantly higher in patients with diabetes compared to healthy controls after cataract surgery^19^. This is also reflected in a study by Le et al., which revealed a significantly higher vessel density in diabetic patients compared to healthy controls after surgery^20^. This might explain the reason why the increase in macular perfusion is most pronounced in the first days after the surgery. However, the persistence over the following months, when the inflammation has mostly subsided, has to be due to other reasons. It is also crucial to consider the administration of anti-inflammatory drugs, as the use of nonsteroidal anti-inflammatory drugs (NSAIDs) and prednisolone eye drops suppresses the release of cytokines to some extent, which limits the ability to accurately measure the true impact of cataract surgery on the posterior segment^21^.

Another factor with the potential to influence retinal vasculature is the IOP. It is widely recognized that IOP decreases after cataract surgery, which is also observed in our study population. This reduction is primarily attributed to the difference in lens thickness between the crystalline lens and the intraocular lens (IOL). With the thinner IOL, the anterior lens capsule assumes a more posterior position, resulting in traction on the ciliary body, with the zonula fibers expanding the lumen of the Schlemm canal^22^. Furthermore, the trabecular meshwork itself undergoes dilation during the surgical procedure. In our study, only the VPI showed a significant correlation to the IOP change, indicating that there are subtle caliber changes occurring after the surgery, possibly due to the reduction in mechanical forces from the IOP on the vascular network. The SVP might remain unaffected, as the larger caliber vessels found there may be less susceptible to the pressure change. The other objective metrics seem unaffected by the IOP, as is the case in the literature^3,5^. Therefore, other factors must play a role in their increase.

In recent years, a new theory has gained traction, suggesting that the elevation in macular perfusion is a manifestation of functional hyperemia, resulting from an increase in retinal metabolism. As cataracts progress, the total amount of light which reaches the retina decrease by up to 40%^23^. When the cloudy crystalline is replaced with a clear IOL, more light is transmitted, which is why patients report discomfort in bright situations the first weeks following cataract surgery. This increase in light leads to an upregulation of the retinal metabolism, which necessitates more oxygen, leading to a rise in blood vessel caliber^18,24,25^. In the literature, it was shown that the increase in retinal thickness correlated significantly with the cataract density, underlying this theory^8,9^.

To the best of our knowledge following a systematic PubMed, Google Scholar and Web of Science search, no prior publication has linked the degree of lens opacification to quantitative changes in retinal vasculature after cataract surgery. The current work demonstrated a significant correlation between the cataract density and the increase in macular perfusion metrics. This correlation was strongest when analyzing the lens nucleus, which plays the primary role in reducing light transmission, while cortical cataracts primarily cause forward light scattering^26^. Meanwhile, no significant correlation to the CDE was established. However, in cataract surgery, the ultrasound energy used for phacoemulsification leads to the creation of hydroxy radicals, which are the main source of the postoperative inflammation^27^. Therefore, we can assume that inflammation only plays a side role, and that the increase in retinal perfusion is due to functional hyperemia responding to the improved light transmission. The increase in vessel density was the most pronounced in the SVP, followed by the DCP and ICP. This could have been expected, as the SVP distributed the blood to the ICP and DCP, with the DCP tasked with supplying the energy demanding photoreceptors^28^. The photoreceptors, requiring more oxygen after surgery, induce vasodilatation and proliferation in the DCP. To meet the increased demand of the DCP and ICP, the SVP undergoes vasodilatation to improve its throughput. Nevertheless, this mechanism remains a postulation, therefore further studies are required to explore how the retinal metabolism adapts following cataract surgery.

All this raises the question of the extent to which the increase in foveal vasculature impacts macular health in both the short and long term. The pseudophakic cystoid macular edema (PCME or “Irvine Gass Syndrome) occurs around 4 to 6 weeks in 0.1–2.35% of patients after cataract surgery^29^. In a study by Chetrit et al., the mean vessel density was significantly lower in patients with PCME compared to healthy control eyes, with the difference being most pronounced in the DCP^30^. The values returned to the healthy level upon resolution of the edema, with these findings being confirmed by later studies^31,32^. Wherever the decrease of vessel density in the DCP is due to a reduction in blood flow, compression by the edematous retinal tissue or distension of the capillary network by the cystoid spaces is still unclear. Nevertheless, the integrity of the DCP is a predictor for the treatment’s effectiveness^32^. Future studies are required to assess how cataract progression impacts retinal vasculature, as a reduction of vessel density due to advanced lens opacification could increase the risk of PCME formation. In that case, early cataract extraction with following functional hyperemia might serve as a protective measure against early complications such as the PCME.

The past decade has witnessed a growing debate regarding a potential association between cataract surgery and Age-Related Macular Degeneration (AMD). While some studies, such as the Beaver Dam Eye Study and the Blue Mountains Eye Study, suggest a higher risk of AMD in individuals who have undergone cataract surgery^33,34^, others, like the Age-Related Eye Disease Study, have failed to establish a clear link between the two^35^. The literature describes a significant decrease in vessel density across all retinal plexuses in AMD patients compared to healthy controls^36^. Further, it is stipulated that altered retinal hemodynamics, through effects on local shear stress and perfusion gradients, create microenvironmental conditions favoring AMD pathogenesis^37^. Population-based studies reveal complex associations between vessel caliber and AMD risk. The Singapore Malay Eye Study demonstrated that wider venular caliber was independently associated with early AMD development, potentially reflecting shared pathogenic processes involving inflammation, dyslipidemia, and endothelial dysfunction^38^. Conversely, the Age-Related Eye Disease Study found lower arteriole-to-venule ratios associated with AMD severity, with narrower arterioles particularly evident in geographic atrophy^39^. To what extent the postoperative hyperemia plays a role in the formation and/or progression of AMD needs to be explored further.

This study is not without its limitation. As only one eye of each patient was included in this work, it is not possible to compare changes in vascular metrics between the fellow eyes. It can be expected that the changes are similar, however, it is unclear how much can be subject to random fluctuations. This could have been explored further through the comparison of the fellow eye. Due to the inherent optical nature of OCTA, we are currently constrained by the density of cataracts, as the image quality deteriorates in lenses with high cataract opacity. Therefore, we cannot make any statement about the change in retinal vasculature in very advanced cases. This will be mitigated by technological advancement accelerating capture times, enabling a higher amount of image averaging, thus improving image quality. Further, the scientific community has urged caution when comparing OCTA images when media opacities change, as variations in the image quality can confound the analysis^40^. However, all images analyzed in this study had a sufficient image quality index even before cataract surgery, and the quality index remained unchanged in the follow up examination. Additionally, the use of image filters and adaptive thresholding techniques in our binarization workflow further reduce the risk of quality induced confounding. Further, many metrics were calculated using the skeletonized map, which is even more robust to quality fluctuations. Consequently, perfusion changes demonstrated here were unlikely the result of the improvement of the ocular optics after cataract removal. Additionally, the longer near infrared wavelength implemented by the SD-OCT has a higher transmission through opacified media compared to visual light, reducing the impact of cataracts on OCT image quality^41^. Lastly, what must be addressed when performing imaging-based research is that differences between individual devices and manufacturers cannot be ruled out. Therefore, having a standardized approach for OCTA acquisition and quantification is of high interest to improve comparability.

## Conclusion

Cataract surgery leads to an increase in macular perfusion which can quantified using multiple objective metrics. This increase correlates significantly with the cataract density quantified using AS-OCT, further supporting the theory that the additional light transmission of the clear IOL leads to a functional hyperemia, and that inflammation and change in IOP are not alone responsible for this change. Further research on the physiological change in retinal metabolism after cataract surgery and the impact this perfusion improvement has on short and long-term compilations is needed.

## Data Availability

All data generated or analysed during this study are included in this published article.
